# Synthesis, Microstructure Investigation, Mechanical and Tribological Behaviour of the AA5083–WC Composite

**DOI:** 10.3390/ma16072891

**Published:** 2023-04-05

**Authors:** Hany R. Ammar, Subbarayan Sivasankaran, El-Sayed M. Sherif, Fahad A. Almufadi, Abdel-baset H. Mekky

**Affiliations:** 1 Department of Mechanical Engineering, College of Engineering, Qassim University, Buraydah 51452, Saudi Arabia; 2 Center of Excellence for Research in Engineering Materials (CEREM), Deanship of Scientific Research, King Saud University, Riyadh 11421, Saudi Arabia; 3 Department of Physics, College of Science and Arts El-Meznab, Qassim University, Buraydah 51931, Saudi Arabia

**Keywords:** AA5083–matrix composite, tungsten carbide (WC), powder metallurgy, microstructure characterization, mechanical properties, tribological behaviour

## Abstract

In this study, AA5083–WC composites were developed by ball milling followed by hot consolidation. The microstructures of the developed composites were investigated using XRD, SEM, EDX, and EBSD. The developed composites exhibited a homogeneous dispersion of WC particulates in the AA5083 matrix without any interactions at the matrix/reinforcement interface. The results confirmed the development of a refined equiaxed grain structure of AA5083–WC composites where the EBSD results revealed an average grain size of 4.38 µm and 3.32 µm for AA5083–6%WC (AW-6) and AA5083–12%WC (AW-12) composites, respectively. The results showed that incorporating WC particulates in the AA5083 alloy matrix significantly improved the compressive stress–strain behaviour and considerably enhanced the resistance to wear and friction. The AA5083–12%WC (AW-12) composite displayed the maximum strength and the highest resistance to wear and friction, whereas the as-milled AA5083 alloy (AW-0) exhibited the lowest strength and the least resistance to wear and friction. The AA5083–12%WC (AW-12) composite exhibited the optimum mechanical and tribological behaviour of the developed composites, making it a promising candidate for tribological applications.

## 1. Introduction

The AA5083 aluminium alloy displays interesting properties, including high strength, lightweight, considerable toughness, high resistance to corrosion and machinability. The AA5083 alloy is promising for several applications such as aerospace, marine, defence and automotive applications. On the other hand, the tribological behaviour of this alloy needs improvement to meet several application requirements, such as high wear resistance and low friction coefficient, because these alloys are subject to relative motion under in-service conditions. AA5083 metal matrix composites (MMCs) have been developed to enhance the tribological behaviour of this alloy for engineering applications. AA5083 MMCs display several interesting mechanical and physical properties such as considerable strength, low density, significant corrosion resistance, considerable fatigue strength, and improved friction and wear behaviour. Numerous studies have reported the incorporation of several reinforcements to aluminium alloys to improve the tribological characteristics and surface properties such as B_4_C [1,2,3], Al_2_O_3_ [4,5], SiC [6,7,8], TiC [9], TiO_2_ [10], and ZrO_2_ [11]. Many techniques have been used to fabricate Al-based metal matrix composites such as powder metallurgy, casting technology, and friction stir processing (FSP) [12]. Nieto et al. [1] studied the influence of B_4_C addition and size on the wear behaviour of AA5083 alloy manufactured using ball milling. It was reported that the hardness increased with decreasing size of B_4_C to the nanoscale where a 56% improvement in the hardness was reported as compared to the AA5083 alloy without B_4_C addition. The wear behaviour of the AA5083–B_4_C nanocomposite also revealed an improvement of approximately 7% over that of the AA5083 alloy without B_4_C addition. Yuvaraj et al. [2] investigated the wear behaviour of AA5083 nanocomposites using mono/hybrid addition of B_4_C/TiC via FSP. They reported that AA5083–B_4_C nanocomposites displayed a hardness of 127 HV and a strength of 364 MPa. Incorporating hybrid B_4_C/TiC nanocomposite resulted in the lowest wear resistance. Mostafapour and Khandani [4] studied the wear of a graphite/Al_2_O_3_ hybrid nano-reinforcement AA5083 alloy processed via FSP. They reported that the optimum combination of wear behaviour and mechanical properties was attained with a hybrid ratio of 50%. Bathula et al. [6] studied the wear of AA5083–10wt.%SiC nanocomposites produced by powder metallurgy route. They reported a significant improvement in the produced composite where AA5083–10 wt.%SiC nanocomposites revealed a hardness value of 280 HV. The friction coefficient and wear rate of AA5083–10 wt.%SiC nanocomposites revealed a substantial improvement as compared to the same properties of the base AA5083 alloy. Mirjavadi et al. [10] examined the influence of TiO_2_ nanosize particles on the wear of AA5083 alloy processed via FSP. They reported a 22% improvement in hardness and strength was achieved and a 16% enhancement in wear resistance for the fabricated AA5083/TiO_2_ nanocomposites. Yuvaraj et al. [13] examined the tribological properties of AA5083/B_4_C nanocomposites fabricated via FSP. The fabricated composite material exhibited improved hardness, strength and wear behaviour compared with the AA5083 base material. Rana et al. [14] studied the tribology of AA5083 base composites with different micron/nano size content of SiC particles manufactured by stir casting. It was reported that the use of SiC nanoparticles resulted in improved tribological behaviour when applying small loads and short sliding distances. On the other hand, the use of microscale SiC particles resulted in improved tribology when increasing the loads to 30 N and sliding distance to 1885 m. Idrisi et al. [15] reported a statistical analysis of the wear behaviour of AA5083–(0, 1 and 2 wt.%) SiC nanocomposite manufactured by stir casting. It was reported that the SiC content had a major effect on the wear resistance of the fabricated composites compared to all the parameters examined in this study. Singh et al. [16] examined the wear of AA5083–B_4_C composites produced by stir casting with B_4_C contents in the range of 5–20 wt.%. Several parameters were examined such as the sliding distance (1000-to-3000 m), load (30-to-50 N) and sliding velocity (1-to-3 m/s). The main conclusion of this study was that increasing the content of B_4_C resulted in improved wear resistance under all examined conditions.

Tungsten carbide (WC) was selected for the present study to improve the tribological behaviour of the AA5083 alloy because it is considered one of the hardest known materials for scientists. WC exhibits interesting properties, such as high strength, stiffness, superior wear resistance, and low coefficient of friction, making it an attractive candidate for wear resistance applications particularly at elevated temperatures [17,18]. AA5083–WC was synthesized using a powder metallurgy solid state route which involved ball milling the AA5083 matrix with various levels of WC particles (0, 3, 6, 9, and 12 wt.%) followed by hot consolidation. This technique provided a homogeneous dispersion of the WC particulates in the AA5083 matrix and minimized the matrix–particulate interaction because the entire fabrication process was carried out in the solid state. The current study aims to (i) develop AA5083–WC composites with different reinforcement contents; (ii) analyse the microstructure of the developed composites using advanced tools such as XRD, SEM, EDX, and EBSD; and (iii) evaluate the mechanical properties of the fabricated composites, including compressive stress–strain and wear/friction behaviours.

## 2. Materials and Methods

### 2.1. Synthesis of AA5083–WC Composites

The as-received powders of the AA5083 alloy and WC were used for ball milling (BM) to synthesize the AA5083–WC composites. AA5083 alloy powders displayed an average size of −325 mesh (44 µm) and WC powders exhibited an average size of −100 + 270 mesh (53–149 µm) with 99% purity. Table 1 shows the standard and actual compositions of the AA5083 alloy. Table 2 displays the intended compositions of the processed composites in terms of volume and weight fractions.

Pulverisette 5/2 classic line ball milling was used to process AA5083–WC composite powder. The as-received AA5083/WC powders were mixed according to the intended composition listed in Table 2. The powders were charged in two vials made from WC (each vial, 250 mL volume). Grinding balls made of WC (10 mm in diameter) were used. The mixed powders were subjected to BM with the following milling variables: 5 h, 250 rpm, and 10:1 balls-to-powder mass ratio (BPR). Dry BM was carried out in an argon atmosphere to prevent oxidation of the processed powders. A total of 2 wt.% of stearic acid was charged with the mixed powders to avoid/minimize cold welding [19,20]. BM was performed for 5 h based on a repetitive cycle of 4 stages, 15 min each, to consist of milling/stop/milling in the reverse direction/and stop. The stop in each milling cycle was considered to reduce heat accumulation inside the vials. Furthermore, forward/reverse milling was applied to ensure the homogeneous dispersion of WC powders in the AA5083 matrix. Moreover, the process input parameters were selected to minimize the possible reaction between the C particles and AA5083 matrix.

The ball-milled powders were sintered using the forging–sintering technique. The developed composite in powder form was heated to 550 °C for 30 min and then hot-forged at 225 MPa using a hydraulic press; the load was maintained for 3 min for proper densification. Powder consolidation was performed in an H13 tool steel die with an internal diameter of 15 mm. Bulk composite samples with a diameter of 15 mm were obtained from this forging–sintering process.

### 2.2. Microstructure Examination of the Developed AA5083–WC Composite

The microstructures of the developed composites were examined by XRD, SEM, EDX, and EBSD. The XRD (Empyrean, Malvern Panalytical, Malvern, UK) investigation was used to identify the phases of the developed composites. In the XRD analysis, Cu-kα was used to examine the samples at a rate (0.6°/min) and step (0.01°) in the 2ϴ range of 20° to 90°. The results obtained from the XRD test were analysed using X’Pert High Score Plus (version 2.2b (2.2.2)). Detailed microstructure analysis was conducted using an electron microscope where an Apreo field emission gun high resolution scanning electron microscope (Apreo FEG-HR-SEM, 30 keV, 1.3 nm resolution at 1 keV, Thermo Fisher Scientific Inc., Waltham, MA, USA) equipped with secondary electron (SE), backscattered electron (BSE), and energy dispersive X-ray (EDX) detectors was used for microstructure analysis. FEG-HR-SEM was used to characterize the size, morphology, and elemental composition of the as-received powders and the processed composites. Furthermore, FEG-HR-SEM was applied to investigate the WC reinforcement dispersion in the AA5083 matrix in both the powder and bulk samples. The worn surface was examined using scanning electron microscopy (SEM), model TESCAN-VEGA3. The FEG-SEM (Lyra3, Tescan, Brno, Czechia) was equipped with electron backscattered diffraction (EBSD). This technique was used to characterize the grain structures of the developed composites. For EBSD analysis, composite samples were prepared for this examination through grinding and polishing procedures where grinding was conducted using SiC sandpapers with different grit sizes. Subsequently, polishing was performed by applying an Al_2_O_3_ solution. Finally, the samples were subjected to Keller’s etchant (95% distilled water, 2.5% HNO_3_, 1.5% HCl, and 1% HF) for 20 s.

### 2.3. Compression and Wear Tests of the Developed AA5083–WC Composite

The developed composite samples with standard dimensions (15 mm diameter × 18 mm height) were subjected to compression tests employing a universal testing machine (MTS Corporation, Eden Prairie, MN, USA) with a crosshead speed at 1 mm/min. The machine was linked with a data acquisition system using Test-works software. Three samples were tested for each composite composition and the average was considered for the present analysis. The load/extension raw data obtained from the MTS machine were used to generate compressive stress/strain curves and the mechanical properties were investigated.

A pin-on-disk machine was used to perform the sliding wear test to study the tribological behaviour of the developed composites. The process parameters of the wear test were a load of 10 N, sliding distance of 2000 m, sliding velocity of 3 m/s, sliding time of 11 min, and a disc rotational speed of 440 rpm. Composite samples with a diameter of 15 mm were polished before applying the wear tests. The wear rate and coefficient of friction of the tested composites were calculated from the results obtained from the wear test using the following equations [21,22]:

Volume loss (mm^3^) = [mass loss (g)/density (g/cm^3^)] × 1000 (1)

Wear rate = volume loss/sliding distance(2)

Coefficient of friction = friction force (N)/applied load (N)(3)

**Figure 1:**
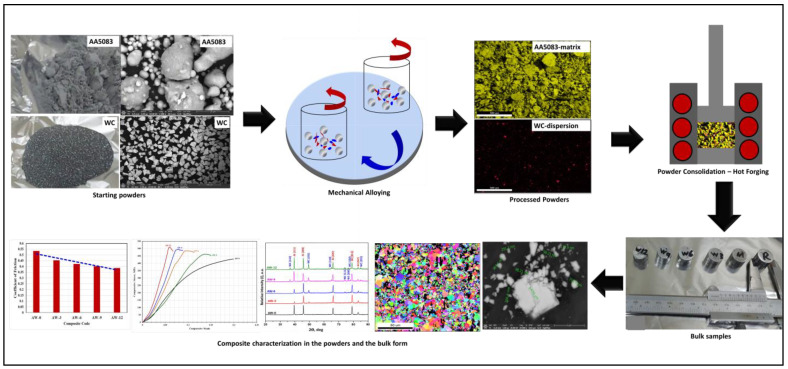
A schematic representation of the main stages of experimental work performed in the present study: starting powders, mechanical alloying, compaction and consolidation, and examination of the microstructure and mechanical behaviour of the fabricated bulk composites.

Figure 1 displays a schematic representation of the main stages of the experimental work performed in the present study including the starting powder, mechanical alloying/ball milling, powders consolidation, examination of the microstructure, and evaluation of the mechanical behaviour of the fabricated composites.

## 3. Results and Discussion

The current study aimed to fabricate AA5083–WC composites with different levels of WC reinforcement (0, 3, 6, 9, and 12 wt.% WC). A solid-state synthesis technique using ball milling was used to ensure the homogenous dispersion of the WC reinforcement in the AA5083 matrix and to avoid any possible reaction between the matrix and reinforcement. A detailed microstructural analysis was performed for both the processed powders and bulk samples. The tribological behaviour and compressive stress–strain behaviour of the fabricated composites were evaluated.

### 3.1. Microstructural Examination of the AA5083–WC Composites

**Figure 2:**
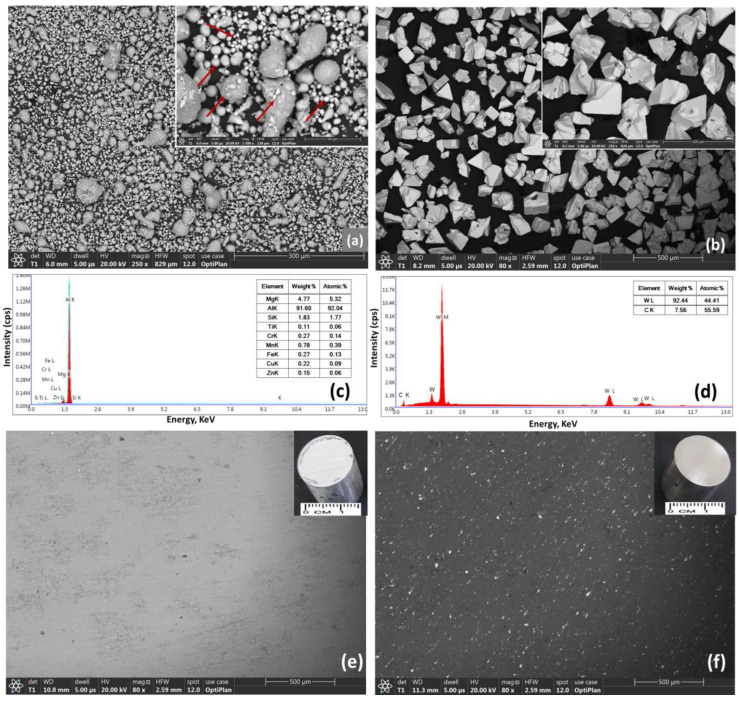
SEM results of the as-received powders and the developed bulk samples: (**a**) BSE of AA5083 powders at low and high magnification; (**b**) BSE of WC powders at low and high magnification; (**c**,**d**) the EDX spectrum of AA5083 and WC powders, respectively; (**e**,**f**) BSE images of AW-0 and AW-6 bulk composites.

The as-received AA5083 alloy powders displayed a particle size of less than 44 μm (<−325 mesh), whereas the as-received WC particles were 53–149 μm (−100 + 270 mesh). FEG-HR-SEM was used to characterize the shape, size, and chemistry of the powders. Figure 2 shows SEM images of the as-received powders and developed bulk samples. Figure 2a displays the backscattered electron (BSE) images of the as-received AA5083 alloy powders, and the inset at the top-right corner displays a higher magnification image of the same AA5083 powders. The as-received AA5083 alloy powders exhibited a spherical morphology with different particle sizes (<44 μm), as indicated by the arrows in the inset of Figure 2a. Figure 2c shows the results of AA5083 alloy powders using the EDX technique to confirm the chemistry of the as-received powders where the EDS results are almost similar to the standard chemical composition shown in Table 1. Figure 2b displays the BSE images of the as-received WC powders, where the insertion at the top-right corner of Figure 2b shows a higher magnification image than the same WC powders. The as-received WC particles exhibited an angular shape with microscale particle size (<149 μm). Figure 2d displays the results of EDX analysis of WC powders which confirmed the chemistry of the received powders where almost equal atomic percentages of W and C are present in the WC particles. Figure 2e shows the BSE image of the as-milled bulk AA5083 alloy without WC addition (AW-0) where the image shows the matrix without any reinforcement particles, and the top-right corner of the same image shows the bulk samples used for this analysis. Figure 2f shows a BSE image of the AA5083–6% WC composite (AW-6) where a uniform dispersion of reinforcement particulates (white particles) was observed on the AA5083–matrix; the insertion in the same image displays the bulk composite sample used for this examination.

**Figure 3:**
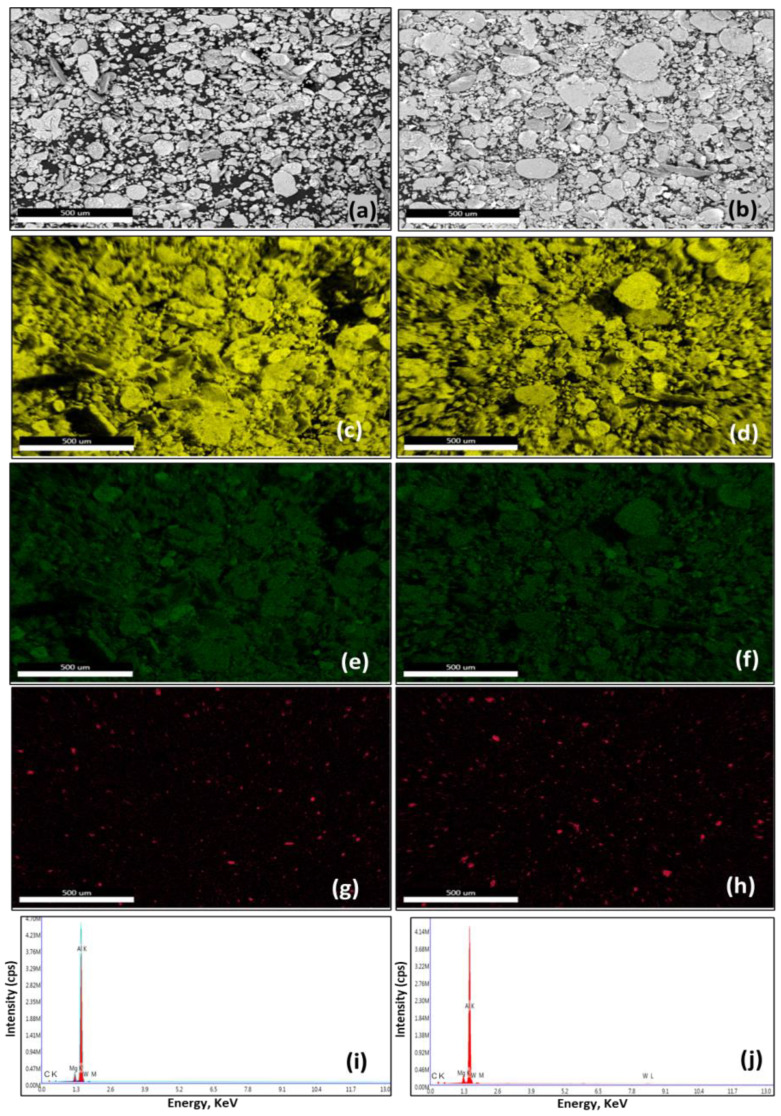
EDX results analysis of the processed powders of AW-6 and AW-12 composites: (**a**,**c**,**e**,**g**,**i**) SE image, Al map, Mg distribution, WC dispersion, and EDX spectrum of the examined area in (**a**) of the processed powders of AW-6 composites, one-to-one; (**b**,**d**,**f**,**h**,**j**) SE image, Al dispersion, Mg map, WC distribution, and EDX spectrum of the examined area in (**b**) of the processed powders of AW-12 composites, respectively.

EDX analysis of the processed powders of the selected composites (AW-6 and AW-12) is presented in Figure 3. Figure 3a,c,e,g,i show the SEM results of the ball-milled powders of the AW-6 composites: (a) SE image; (c) Al map; (e) Mg distribution; (g) WC dispersion; and (i) EDX spectrum of the examined area in the SE image in (a). It should be noted that the elemental mapping represented only the dispersion of the two major elements in the AA5083 alloy, namely, aluminium and magnesium, where Mg is dissolved in Al, as a solid solution in the AA5083 alloy, as may be observed from Figure 3c,e. In addition, the EDX map represents only the dispersion of tungsten (W) instead of (WC) to avoid mapping the carbon from the adhesive carbon tabs used to hold the powder samples for SEM-EDX analysis. Figure 3g shows an even dispersion of the WC particles in the AA5083 matrix. The EDX spectrum presented in Figure 3i conformed to the chemistry of the developed composite where the peaks of the main elements of the alloy matrix (Al and Mg) and reinforcement particles (W and C) were identified. Similar results were observed for the ball-milled AW-12 composites presented in Figure 3b,d,f,h,j with a notable increase in the content of homogenously dispersed WC in the AA5083 matrix in AW-12, as shown in Figure 3h. The results presented in Figure 3 confirm the development of homogenous AA5083–WC composites in the processed powder form where an even dispersion of WC particles were observed in the AA5083 alloy matrix in both the AW-6 and AW-12 composites.

**Figure 4:**
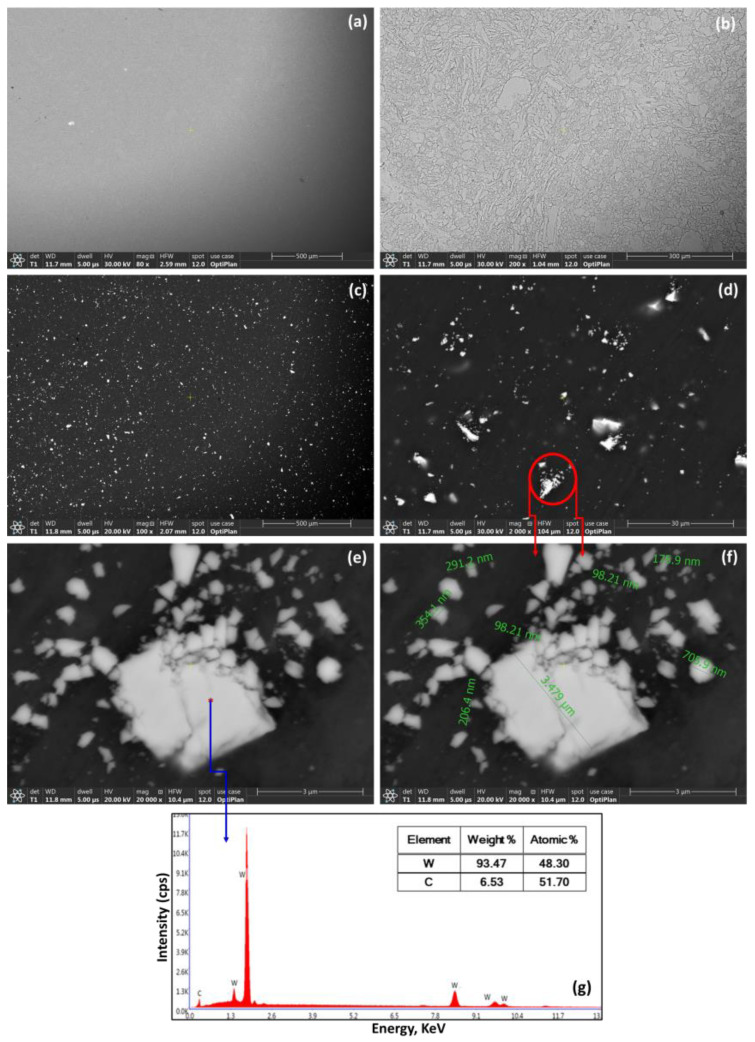
BSE images of the developed bulk solid composites (AW-0 versus AW-6): (**a**,**b**) BSE images of AW-0 at low and high magnification, respectively; (**c**) BSE image of the WC dispersion in AA5083 (AW-6 composite); (**d**,**e**) BSE images of WC fragmentation; (**f**) particle size of the fragments of WC particles; (**g**) EDX spectrum for point indicated in (**e**).

Figure 4 shows BSE images of the developed bulk solid composites where AW-0 and AW-6 are presented to confirm further the effect of ball milling on the dispersion of WC in the AA5083 matrix. Figure 4a,b show the BSE images of AW-0 (AA5083 alloy without reinforcement) at low and high magnifications, respectively, where the matrix is free from any phases/particles. The grain structure of AW-0 was observed at higher magnification without observing the other phases in the matrix. In contrast, Figure 4c shows a homogenous dispersion of WC particles over the AA5083 matrix in the AW-6 composites. The ball-milling technique provided an essential uniform reinforcement dispersion and notable fragmentation of the WC particles, as shown in Figure 4c–e. The ball-milling mechanism of repeated collision of the grinding media (balls and container walls) with the charged powders (in this case: AA5083–6% WC) resulted in repeated fracturing of the WC particles, owing to the brittleness of these particles, as observed in Figure 4d. The circled WC particles in Figure 4d were further investigated at higher magnification to confirm the fragmentation of WC particles where Figure 4e displays the circled particles at high magnification with an EDX spot analysis of the white particles to confirm their chemistry, as shown in Figure 4g where the EDX spectrum confirmed the chemistry of these phases as WC particles. Figure 4f shows the WC particle size where a significant reduction in particle size was observed. The starting particle size of the WC was 53–149 µm. However, in Figure 4f, the size of WC was significantly reduced to ≈4 µm and less, and most of the small fragments of WC particles were observed to reveal a refined size. The ball-milling mechanism offers significant advantages to the developed composites, such as the homogenous dispersion of the WC reinforcement over the AA5083 matrix, a substantial reduction in the sizes of the WC particles, and no interaction between the reinforcement and matrix.

### 3.2. XRD Analyses of the Developed Bulk Composites

**Figure 5:**
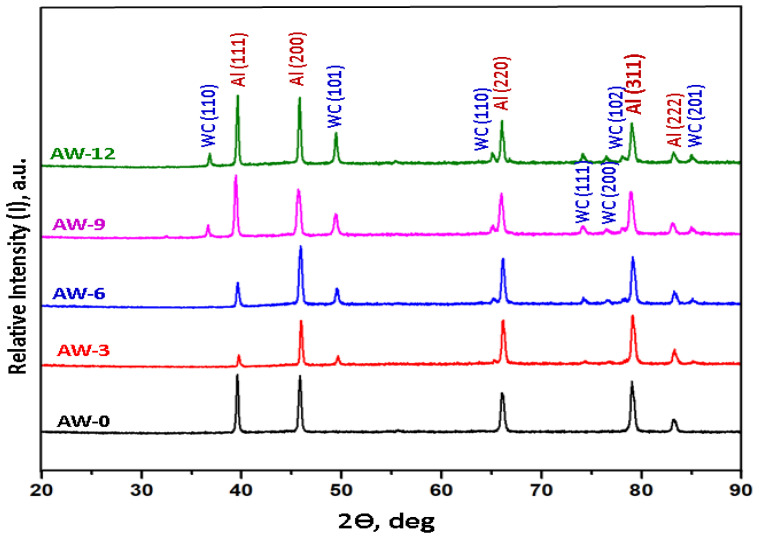
XRD patterns of the developed AA5083–WC bulk composites.

The XRD results of the developed bulk composites are presented in Figure 5 where the XRD patterns show that the predominant indexed phase in the as-milled AA5083 alloy (AW-0) is face-centred cubic aluminium, and the appearance of Al peaks only in the AW-0 sample indicates the presence of the remaining elements in the AA5083 alloy as a solid solution. These observations are consistent with the elemental mapping of the major elements (Al and Mg) in AA5083 alloy presented in Figure 3 and agree with the BSE images presented in Figure 4a,b, where no phases were detected in the matrix. As shown in Figure 5, the XRD patterns of AW-3, AW-6, AW-9, and AW-12 bulk composites revealed two major phases, namely, cubic Al (reference code: 01-089-2837 and space group: Fm-3m, number 225) and hexagonal WC (reference code: 01-073-0471 and space group: P-6m2, number 187). The results of elemental mapping (Figure 3), BSE imaging (Figure 4), and XRD patterns (Figure 5) confirmed that there were no additional intermetallic phases formed in the developed composites. The formation of Al-C/Al-W intermetallic phases is usually created when using traditional melting routes of production because the melting temperature increases the affinity to form such intermetallic phases [23,24]. However, the absence of intermetallic phases in the developed composites was attributed to the solid-state process used to fabricate these composites. The heat generated during powder processing was insufficient to cause the formation of additional intermetallic phases [6,23].

### 3.3. The EBSD Analysis of the Developed AA5083–WC Composites

**Figure 6:**
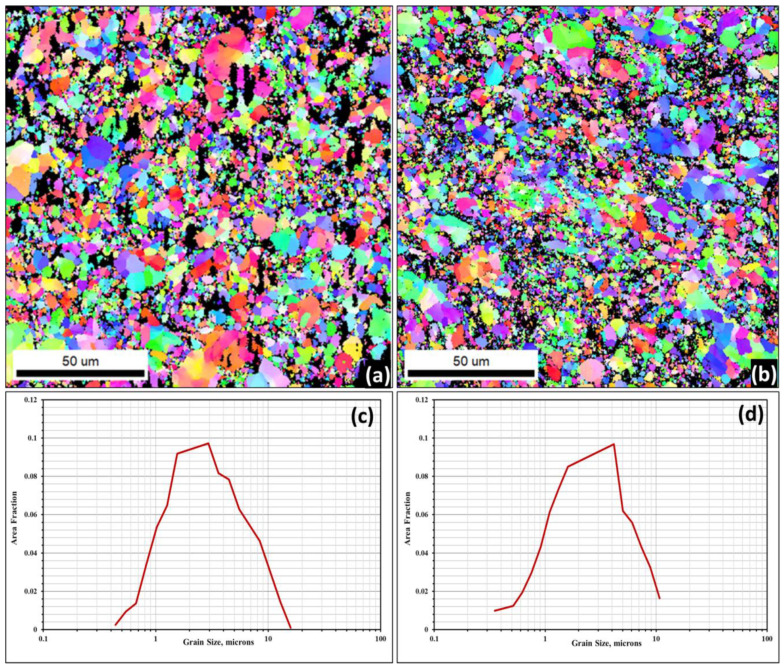
EBSD coloured maps and the corresponding grain size distribution of selected composites. (**a**,**b**) are the inverse pole figure (IPF) coloured maps of AW-6 and AW-12, composites, respectively. (**c**,**d**) are the grain size distribution corresponding to the IPF grain structure map for AW-6 and AW-12 composites, respectively.

Figure 6 displays the EBSD coloured maps and the corresponding grain size distributions of the AW-6 and AW-12 composites, which were selected as samples for this investigation. Figure 6a,b show the inverse pole figure (IPF) coloured maps of the AW-6 and AW-12 composites, respectively. Figure 6c,d show grain size distribution corresponding to the IPF grain structure map for the AW-6 and AW-12 composites, respectively. A fine equiaxed grain structure was observed for the developed bulk composites where a narrow distribution range of the grain size was noted. The determined average grain sizes were 4 µm and 3 µm for the AW-6 and AW-12 composites, respectively. The refined grain structure obtained for the developed composite was attributed to the effect of the ball-milling process on refining the grain structure [25] where both the AA-5083 matrix and WC reinforcement were subjected to repeated fracturing mechanisms during the milling process, which led to a refined equiaxed grain structure, as shown in Figure 6. In addition, dynamic recovery and recrystallization play a significant role in providing the fine equiaxed grain structure of the developed composites, as discussed in the following section.

**Figure 7:**
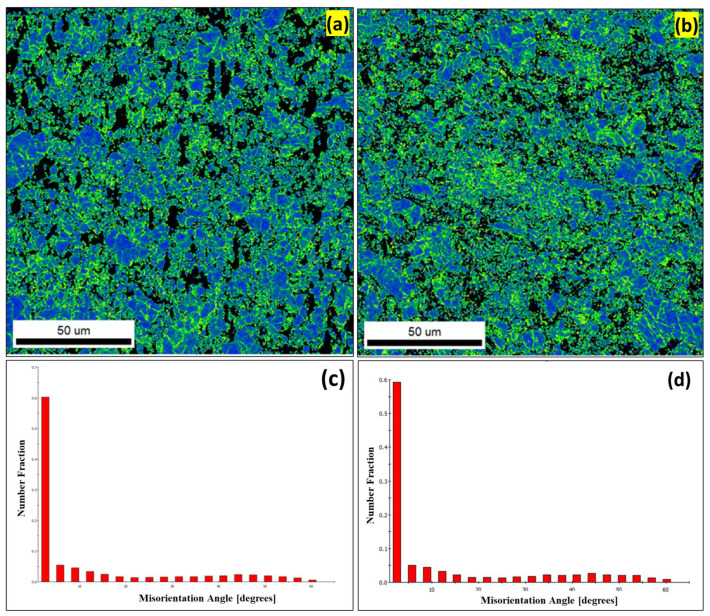
(**a**,**b**) KAM map of AW-6 and AW-12 composites, respectively; (**c**,**d**) the corresponding misorientation angle distribution.

Figure 7 shows the Kernel average misorientation map (KAM) and corresponding misorientation angle distributions for the AW-6 and AW-12 composites. The KAM is used as an indication of a local grain misorientation and provides a quantitative analysis of the average misorientation angles [26]. The KAM reveals higher values for deformed grains because of the higher density of dislocation [27,28]. The KAM results support the understanding of lattice distortions, localized deformation, dislocation density, and stored strain energy. Figure 7a,b show the maps (KAM) of the developed AW-6 and AW-12 composites, where a high dislocation density was observed for the developed composites. Figure 7c,d shows the misorientation angle (the smallest angle between two crystals) distribution where the range of 1° ≤ angle ˂ 5° identifies the sub-grains (SG); 5° ≤ angle ˂ 15° describes the low-angle grain boundaries (LAGBs); and 15° ≤ angle ˂ 180° specifies the high-angle grain boundaries (HAGBs). According to the results presented in Figure 7, the developed composites AW-6 and AW-12 exhibited >70% SG and LAGBs and the remaining exhibited <30% HAGBs. These results confirm the higher dislocation density of the developed AW-6 and AW-12 composites. The high dislocation density indicates lattice distortion and localized deformation resulting from composite processing using ball milling, which produced many lattice imperfections and plastic deformation in the refined equiaxed grain structure. Furthermore, incorporating ultrafine WC particles, as shown in Figure 4f, into the AA5083 matrix resulted in the additional pinning of dislocations, leading to a greater dislocation density, more lattice distortion, and increased stored strain energy. The misorientation angle distribution is shown in Figure 7c,d, revealing low values for a wide range of grains in the developed composites, which refers to grain recrystallization [29]. The existence of SG and LAGBs is a sign of dynamic recovery [24] where dynamic recrystallization occurs through the gradual conversion of LAGBs into HAGBs through dislocation reordering [30,31]. Therefore, dynamic recovery and recrystallization played a significant role in providing the fine equiaxed grain structure to the developed composites.

### 3.4. The Compressive Stress–Strain Behaviour of the Developed AA5083–WC Composites

**Figure 8:**
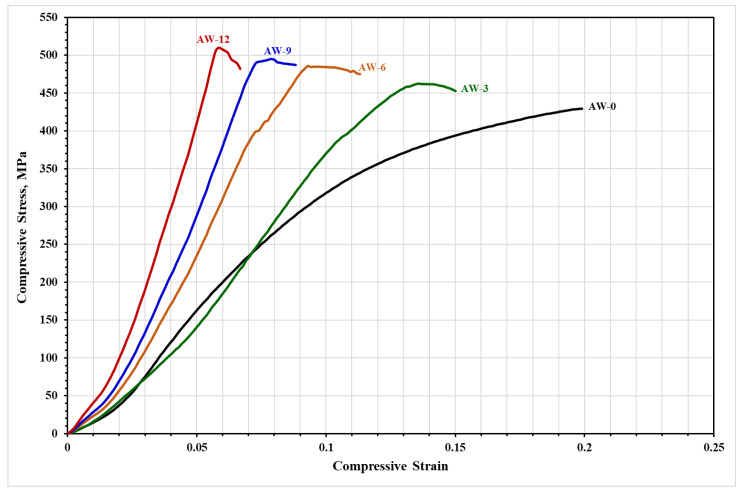
Compressive stress–strain curves of the developed AA5083–WC composites.

The compressive stress–strain curves of the developed composites are shown in Figure 8 and the values of the ultimate compressive stress and the corresponding strain of the developed composites are listed in Table 3. Figure 8 shows a significant increase in the composite strength with an increase in the WC reinforcement content. The AW-12 composite displayed the maximum strength (511 ± 3 MPa) with the least corresponding strain (0.08 ± 0.001), while the as-milled AA5083 alloy (AW-0) exhibited the lowest strength (429 ± 3 MPa) with the greatest corresponding strain (0.2 ± 0.002). The elastic modulus of the developed composites was calculated from the stress–strain curves shown in Figure 8; the values are listed in Table 3. A substantial increase in the elastic modulus of the composites was achieved by increasing the WC content. The AW-12 composite exhibited the maximum stiffness, whereas the as-milled AW-0 composite exhibited the lowest stiffness. A gradual increase in the composite strength at the expense of ductility is observed in Figure 8 with an increase in the WC content. These observations confirm the role of the incorporated WC particles on the mechanical behaviour of the developed composites where increasing the WC content reduced the ductility and enhanced the strength of the composites [32,33]. The improved strength of the developed composites is attributed to several strengthening/hardening mechanisms. The ball-milling process and the incorporation of WC provided several structural imperfections such as lattice strain, dislocation density, and stored strain energy. The developed composites exhibited a refined equiaxed grain structure (Figure 6) which activated grain boundary hardening according to the Hall–Petch concept [34]. Hard WC particles with refined size and homogenous dispersion (Figure 4) effectively carry the applied load and activate the Orowan strengthening mechanism [35]. The AA5083/WC interface displayed superior bonding without any detected interaction between the matrix and reinforcement, which significantly enhanced the strength of the developed composites [24,35,36]. The incorporated WC particles contributed to resisting dislocation motion and increasing the dislocation density, whereas the developed composites displayed a high density of dislocations (Figure 7). These effective hardening mechanisms improve the strength of the developed composites by increasing the content of WC particles [24,34].

### 3.5. Tribological Behaviour of the Developed AA5083–WC Composites

**Figure 9:**
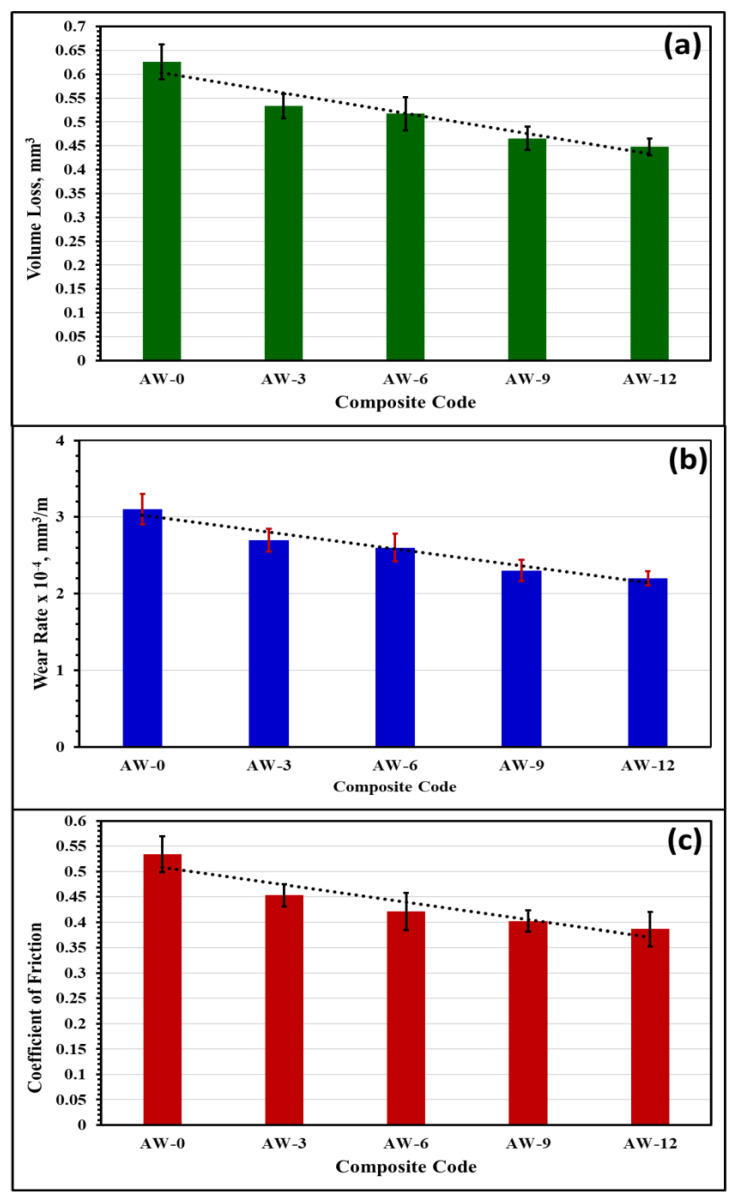
Tribological behaviour of the developed composites: (**a**) the volume loss; (**b**) the wear rate; and (**c**) the coefficient of friction.

Sliding wear tests were performed to study the tribological behaviour of the AA5083–WC composites. The test was performed under a load of 10 N, sliding distance of 2000 m, sliding velocity of 3 m/s, sliding time of 11 min, and disc rotational speed of 440 rpm. The process input parameters of the sliding wear tests were kept constant to display the effect of the WC content on the wear and friction behaviours of the developed composites. Tribological properties such as volume loss, wear rate, and coefficient of friction of the tested composites were calculated from the obtained results according to Equations (1)–(3) [21,22]. Figure 9 shows the tribological behaviour of the developed composites where Figure 9a shows the volume loss, Figure 9b shows the wear rate, and Figure 9c shows the coefficient of friction of the developed composites as a function of the WC content. The volume loss, wear rate, and coefficient of friction were observed to decrease with the increase in WC particle content. The AW-12 composites provided the best tribological behaviour with the minimum volume loss, smallest wear rate, and lowest friction coefficient. On the other hand, the milled AA5083 alloy matrix without reinforcement (AW-0) revealed the highest volume loss, maximum wear rate, and friction coefficient. According to the results presented in Figure 9, the volume loss, wear rate, and coefficient of friction decreased with the increase in WC content in the AA5083 alloy matrix.

Strength has been reported to affect the wear resistance of materials [37,38,39]. The increased strength reduces the volume loss, decreases the wear rate, and minimizes the coefficient of friction. According to Figure 8 and Figure 9, as the strength of the developed composites increased, the wear resistance increased. The optimum wear resistance was attained for the composite with the maximum strength (AW-12), whereas the lowest strength composite (AW-0) exhibited the largest volume loss, maximum wear rate, and greatest friction coefficient. The stiffness (elastic modulus) was found to affect the wear behaviour of the materials [37,40,41]. Table 3 presents the calculated elastic moduli of the composites. According to the results in Table 3 and Figure 9, the wear and friction resistance improved with an increase in the composite stiffness. The increased elastic modulus of the composites reduced the material removal by the pin indenter and minimized the deformation of the tested surface. Accordingly, the increased stiffness resulted in a reduction in volume loss, wear rate, and friction coefficient. The AW-12 composite displayed the maximum stiffness and minimum wear rate and friction coefficient, whereas the AW-0 composites exhibited the minimum stiffness, maximum wear rate, and friction coefficient.

The wear results are consistent with the results displayed in Figure 8 where the hardening mechanisms responsible for the improved strength and reduced deformability of the developed composites resulted in improved wear resistance and friction coefficient according to Archard’s principles, where there is an inverse correlation between the wear rate and material hardness [42,43]. The improved tribological behaviour, strong resistance to wear, and friction of AA5083–WC composites incorporating high contents of WC particles are attributed to the effective hardening mechanisms mentioned in Section 3.4, which improved the hardening behaviour and reduced deformability of the developed composites [6,8,42,43,44,45,46]. The tribological behaviour observed in the present study agreed with previous studies with numerous Al-matrix-reinforcement types such as Al5083-SiC_p_ [6], A356-Al_2_O_3_ [42], A5083-WC-Al_2_O_3_ [43], and Al6061-SiC-Gr [44].

### 3.6. Worn Surfaces Analysis of the Developed Composites

**Figure 10:**
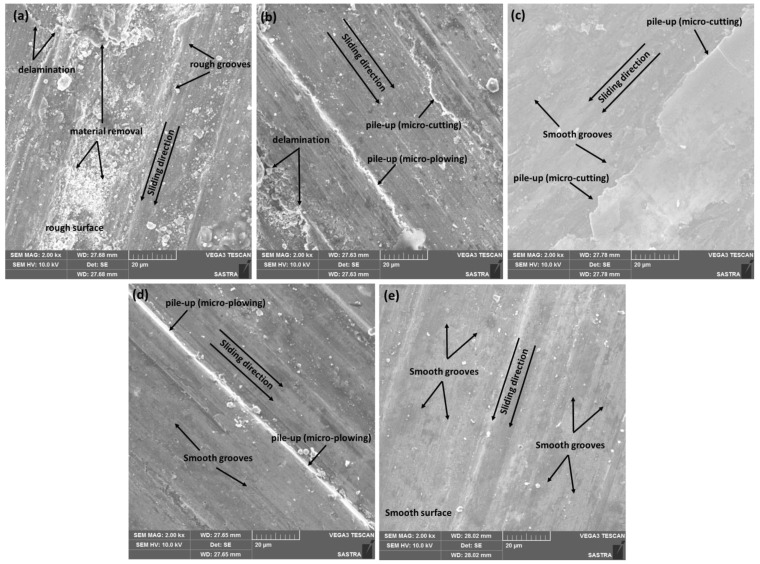
Worn surface morphology of the developed composites: (**a**) AW-0, (**b**) AW-3, (**c**) AW-6, (**d**) AW-9, and (**e**) AW-12.

Figure 10 shows secondary electron images of the worn surface morphology of the developed composites. Figure 10a reveals the topography of the worn surface of the AW-0 sample where rough grooves, delamination, and rough surface features with notable material removal and plastic deformation are observed. According to Section 3.4 and Section 3.5, the AW-0 sample displayed the lowest strength, the highest ductility, the maximum wear rate, and the maximum friction coefficient when compared with the remaining samples. Accordingly, the observations related to its worn surface features support the attained mechanical and tribological behaviour where this sample exhibited low wear and friction resistance and an effective adhesive wear mechanism [43]. Figure 10b displays the worn surface features of the AW-3 composite where an abrasive wear mechanism is effective in the form of micro-ploughing, micro-cutting, and smooth grooving. There is a material pile-up due to this abrasion mechanism [43,46]. The surface seems softer than the AW-0 sample with a smaller delamination area and smoother grooves along the sliding direction. Figure 10c displays the worn surface of AW-6 composite where a smoother worn surface with smooth grooves is observed compared to AW-3 samples. Furthermore, a material pile-up due to abrasive micro-cutting was detected. Based on the topography of the worn surface of the AW-6 composite, improved resistance to wear and friction was achieved as observed from the overall material deformation/removal of the worn surface. Figure 10d shows the worn surface of the AW-9 where the surface topography becomes smoother with fine grooves. In addition, a material pile-up due to abrasion micro-ploughing was observed. Figure 10e shows the worn surface of the AW-12 composite where a smooth surface with fine abrasive grooves along the sliding direction was observed. When comparing the worn surfaces in Figure 10a–e, it may be observed that increasing the WC content reduced the material removal/plastic deformations and provided fine and smooth grooves with an overall smooth worn surface [6,43,46]. Incorporating hard WC particles increased the hardening mechanisms where the strength of the composite increased, and the ductility decreased which improved the composite resistance to wear and friction [6,43,46,47]. The AW-12 composite exhibited a worn surface with the minimum material removal/deformation, which indicated the highest resistance to wear and friction due to its high content of WC particles. The WC particle content played a significant role in improving the strength, hardness, and resistance to wear and friction. These results are consistent with the results presented in Figure 8 and Figure 9, where the AW-12 composite exhibited the highest strength, lowest wear rate, and friction coefficient, whereas the AW-0 exhibited the opposite behaviour. The AA5083 alloy exhibited significant improvements in the mechanical and tribological behaviours by incorporating 12 wt.% WC particles.

## 4. Conclusions

In the present study, the AA5083–WC composites were developed using ball milling followed by hot consolidation. According to the results obtained in the present study, the following conclusions were drawn.

- The developed composites exhibited a homogeneous dispersion of WC particulates in the AA5083 matrix without any interactions at the interface.
- The EBSD results confirmed the development of a refined equiaxed grain structure of AA5083–WC composites with an average grain size of 4 µm and 3 µm for the AA5083–6%WC (AW-6) and AA5083–12%WC (AW-12) composites, respectively.
- The Kernel average misorientation map (KAM) and corresponding misorientation angle distributions for the AW-6 and AW-12 composites illustrated that more than 70% were sub-grains (SG) and low-angle grain boundaries (LAGBs) whereas the remaining (<30%) were high angle grain boundaries (HAGBs).
- Increasing the WC content (0–12%) resulted in a considerable enhancement in mechanical and tribological behaviours of the AA5083–WC composites.
- The wear and friction resistance of the composites were observed to increase with increasing the strength and stiffness of the developed composites.
- The AA5083–12%WC composite exhibited the optimum tribological behaviour with the minimum volume loss, lowest wear rate, and lowest friction coefficient.
- The as-milled AA5083 alloy (AW-0) exhibited the minimum wear and friction resistance with the highest volume loss, maximum wear rate, and friction coefficient.

## Figures and Tables

**Table 1:** The elemental composition of AA5083 alloy.

Element	Al	Mg	Mn	Cr	Si	Fe	Cu	Zn	Ti	O
Standard (wt.%) *	Bal.	4.0–4.9	0.4–1.0	0.05–0.25	≤0.4	≤0.4	≤0.4	≤0.4	≤0.4	≤0.4
Actual (wt.%)	Bal.	4.71	0.7	0.19	0.07	0.16	0.04	0.06	≤0.01	0.19

*wt. % refers to weight percent

**Table 2:** The intended composition of the developed composites in volume and weight fractions.

Composite Code	AA5083		WC	
	wt. fr. *	vol. fr. *	wt. fr.	vol. fr.
AW-0	1.00	1.000	0.00	0.000
AW-3	0.97	0.995	0.03	0.005
AW-6	0.94	0.989	0.06	0.011
AW-9	0.91	0.984	0.09	0.016
AW-12	0.88	0.977	0.12	0.023

* wt. % and vol. fr. refer to weight percent and volume fraction, respectively

**Table 3:** The ultimate compressive stress, corresponding strain, and elastic modulus of the developed composites.

Composite Code	Ultimate Compressive Stress (MPa)	Strain at Ultimate Point	Elastic Modulus (MPa)
AW-0	429 ± 3	0.22 ± 0.002	3725 ± 16
AW-3	462 ± 3	0.15 ± 0.001	4060 ± 9
AW-6	486 ± 2	0.12 ± 0.001	4577 ± 13
AW-9	495 ± 1	0.11 ± 0.001	5563 ± 9
AW-12	511 ± 2	0.08 ± 0.001	7778 ± 11

## Data Availability

The experimental datasets obtained from this research work and the analysed results during the current study are available from the corresponding author on reasonable request.
